# Cesarean Operation Terminating Fatally

**Published:** 1842-05

**Authors:** John Travis

**Affiliations:** Carroll county, Tennessee


Art. II.—*Cesarean Operation terminating fatally.* By John Travis, M. D., of Carroll county, Tennessee.

In February, 1837, I was called to Mrs. P——, a corpulent lady, the mother of seven children, and wife of E. P——, of Carroll county, Tennessee. Her full period of utero-gestation had arrived, and she had been in charge of a midwife 48 hours, suffering the most excruciating pains of labor, without any beneficial effects. On examination I found that the os uteri had not dilated, nor was it susceptible of dilatation. It is true there was an aperture the size of a dime, but this could not be enlarged by any possible effort of nature. I learned that the midwife who delivered her of her last child, had, to use the language of my informant, *torn her to pieces.* The uterus had been ruptured, the bladder injured, and the vagina and its appendages so lacerated, that her life was saved with great difficulty, and her health was only restored after being in the charge of a physician and good nurse for five months. She could not retain her urine, it passed off by drops, keeping her linen constantly wet. In fact she had been ruined by the midwife, and from a careful examination it appeared extraordinary that she became pregnant after having sustained so serious an injury. The patient herself expressed astonishment at the event, as penetration could be effected only to a very small extent.

Having never performed the Cesarean operation, and knowing its fatal tendency, I was unwilling to attempt it alone, and sent for Dr. Moore, then of Huntingdon, to assist me. On his arrival he made an examination, and ascertained that there was no chance to deliver the child without an operation, but expressed his unwillingness to undertake it, and informed the patient, as I had done previous to his arrival, that such operations had generally failed; that in England nine cases out of ten proved fatal, &c. But she urged us to operate, saying that she would have it done if she was certain that death would supervene while she was under the knife. She said her sufferings had become insupportable, and her friends likewise urged us to operate.

Being thus importuned, we finally determined to perform the operation, knowing that the life of the child might be preserved, and that there was a remote probability of saving the life of the mother.

The woman was now placed on a narrow bed, on her back, and everything being ready, I made an incision in the abdominal teguments with the scalpel, from the umbilicus, to within an inch of the symphisis pubes; then the linea alba was divided, next the peritoneum, and finally the uterus. The child being removed, the wound was closed with sutures, adhesive strips, &c. The patient said she was easy, and that she did not suffer much pain from the operation.

She was put under a rigid antiphlogistic treatment, and for four or five days appeared to be doing very well. The lochial discharge passed as after a common delivery. About this period, in spite of our entreaties, she sat up and walked about in the house. On the sixth or seventh day she complained of a burning in her stomach and abdomen, vomited incessantly, had high fever, and died on the eighth day. Her child is a fine boy and is still living.

In connection with this case, I cannot help remarking that it is a matter of sincere regret, that there is no statute to prohibit ignorant women from the practice of midwifery. The necessity of the operation just mentioned, was, no doubt, caused by the ignorance and rashness of a midwife in a former pregnancy. I have lately seen another woman who was delivered of a child ten years ago, and she was injured like Mrs. P. She has had no catamenial discharge for ten years, nor any intercourse with her husband.

January, 1842.

